# Movement Ecology and Disease Exposure in Free-Roaming Donkeys in California, USA

**DOI:** 10.3390/ani16081269

**Published:** 2026-04-21

**Authors:** Sarah R. B. King, Amy McLean, Jacob D. Hennig, Kathryn A. Schoenecker

**Affiliations:** 1 Natural Resource Ecology Laboratory, Warner College of Natural Resources, Colorado State University, and in Cooperation with the U.S. Geological Survey, Fort Collins Science Center, Fort Collins, CO 80526, USA; 2 Animal Science, University of California Davis, Davis, CA 95616, USA; 3 School of Natural Resources & the Environment, University of Arizona, Tucson, AZ 85721, USA; jhennig@arizona.edu; 4 U.S. Geological Survey, Fort Collins Science Center, Fort Collins, CO 80526, USA; schoeneckerk@usgs.gov

**Keywords:** burro, Death Valley, Mojave Desert, habitat use, space use, asinine herpesvirus, *Streptococcus equi*

## Abstract

Feral donkeys (*Equus asinus*) are well adapted to arid ecosystems and are found in large populations in the deserts of Australia and the Americas. We assessed resource selection and seasonal home range size of female donkeys in southern California between 2020 and 2022 based on telemetry data. We also examined whether dyads with greater encounter rates were more likely to test positive for asinine herpesvirus 5 (AHV-5) and/or *Streptococcus equi zooepidemicus* (SEZ). Donkey home ranges were non-significantly larger in the cool/wet season (November through March; mean 318.37 ± sd 417.65 km^2^) than in the hot/dry season (April through October; mean 159.35 ± 212.43 km^2^). Donkeys selected flatter areas closer to water year-round but selected greater herbaceous cover during the cool/wet season and lower heat loads during the hot/dry season. Individuals testing positive for SEZ selected lower elevations during the wet season and closer distances to water during the dry season; donkeys testing positive for AHV-5 selected areas farther from water during the wet season and steeper slopes during the dry season. The dyad encounter rate was unrelated to presence of either disease. Our results contribute to the understanding of donkey ecology, allowing feral populations to be better controlled by specific and focused management.

## Simple Summary

Donkeys survive well in the deserts of Australia and the Americas without human care, meaning that their populations can grow quickly. With little predation, the only limits that stop populations from increasing are food availability and disease. We studied feral donkeys in the Mojave Desert of southern California between 2020 and 2022 using radio collars. We looked at how far they moved and their space use, exploring whether animals that were frequently close to each other would share disease. We found that donkey home ranges tended to be larger in the cool/wet than the hot/dry season, and that although they always selected flatter areas close to water, they made other habitat choices depending on season. Donkeys infected with different diseases selected habitats differently. Understanding how donkeys use the range and how they are affected by disease is helpful in informing management of feral populations inhabiting arid regions.

## 1. Introduction

There is an inextricable link between a species’ spatial and social behavior and how disease is transmitted among individuals [1]. A higher disease transmission rate would be expected among gregarious species [2], but this risk can be mitigated through social network organization [3,4] and the spatial structure of social contact events [5]. Knowledge of how animals move and aggregate can therefore reveal important information about their ecology, not just in terms of habitat use but also in terms of how disease can spread within a population. Models of potential disease spread can be improved by incorporating individual-based data [6] due to variation in the role of individuals in disease dynamics and because individuals assort non-randomly [6,7]. Stable social groups that are organized spatially are likely to have smaller epidemics [7].

Ungulate aggregations are largely determined by distribution of resources, with females remaining more clustered where resources are widely available and being more dispersed where resources are limited in spatial scale [8]. When free-roaming in a feral state, donkeys (*Equus asinus*) demonstrate the same social system as their progenitor species, the African wild ass (*E. africanus*), which evolved in the deserts of North Africa [9]: in arid regions of the United States, donkeys have no permanent social bonds between individuals, except for mother and recent offspring [9,10]. Individuals therefore come together and split apart in a fission–fusion social system [11], congregating or meeting other individuals for mating or for access to resources such as water holes. When individuals meet, they can share information about familiarity, health, and reproductive status through sampling odor directly or through fecal marking [12,13,14], and they also have the potential to pass on disease [2].

Donkeys are not native to the United States and were introduced by colonists at a similar time to horses (*E. caballus*) [15]. In addition to animals held as livestock, there are currently more than 19,000 free-roaming donkeys in areas managed by the U.S. Department of Interior Bureau of Land Management [16], with similar numbers on military and National Park Service properties [15]. Donkey diets reflect availability in the habitat, being less diverse and consuming more browse in desert compared to relatively more mesic habitats [17]. Free-roaming donkeys select plants depending on their most nutritious growth stage, consuming browse in the winter and otherwise mostly graminoids and forbs [18,19]. In the deserts of the southwestern United States, feral donkeys’ home ranges are reported between 35 km^2^ and 254 km^2^ [20,21]. The effect of season on range size varies across populations; however, a consistent pattern is for donkeys to select areas offering more resources in terms of water availability or herbaceous vegetation or habitats where they can protect themselves from solar radiation [20,21,22]. In southwestern deserts, feral donkeys can affect vegetation through consumption and trampling and subsequently alter habitats of resident small mammal and bird communities [23].

Disease in donkeys is under-studied in comparison with that in horses [24]. Ass and horse lineages are phylogenetically separated by 2.1 million years [25], resulting in differences in horse and donkey physiology, such as a narrower nasal cavity and smaller pharynx in the donkey [24]. The relatively stoic nature of donkeys means that infection may be inapparent until it reaches an advanced stage [26,27]. Donkeys can be more severely affected by equine influenza than horses and are more susceptible to glanders [27]. Like horses, donkeys can be infected with herpes viruses, including equine and asinine herpes viruses (EHV and AHV). Herpesviruses are DNA enveloped viruses [28] that can establish chronic infections and cause severe disease [29]. These viruses can persist in populations by remaining latent in asymptomatic hosts [30] until stressors or hormonal changes reactivate the virus and it resumes shedding [29,31]. Asinine herpes virus 5 (AHV-5) is closely related to the wild ass herpes virus (WAHV) and is distinctly different phylogenetically from EHV-5, although both are in the same gamma herpesvirus subfamily [32].

In donkeys, AHV-5 has been associated with respiratory and neurological diseases, with infected individuals also suffering from oxidative stress and tissue damage [27,28,29,33]. Clinical signs of AHV-5 can range from mild rhinitis to interstitial pneumonia [27]. Respiratory diseases in donkeys can be aggravated due to prevalence of hyperlipidemia in this species [28] and can be fatal [31]. Herpesviruses can be spread by aerosol transmission from nasal discharge and have been found to be stable and infectious in water sources used by wild equid species [34]. Donkeys can be coinfected with AHVs and *Streptococcus equi* subsp. *zooepidemicus* (SEZ) [27]. *Streptococcus equi zooepidemicus* is transmitted when individuals are in close proximity. Water sources are a potential infection point for equids: during drought, animals are likely to be stressed due to aggregation at a limited resource, resulting in higher susceptibility to disease [33].

*Streptococcus equi zooepidemicus* is a beta-hemolytic streptococcus belonging to the Lancefield group C [35]. It is a commensal bacterium of the oral cavity, pharynx, and respiratory tract of equids and can also affect the reproductive tract. *Streptococcus equi zooepidemicus* can be an opportunistic pathogen following other disease infections or stress [26]. While it can be present in healthy animals with no clinical signs (potentially due to donkeys’ insensitive cough reflex compared to horses [36]), it has the potential to cause respiratory diseases, including pneumonia and strangles-like diseases [26]. *Streptococcus equi zooepidemicus* can also be a causative agent of other infections, such as endometritis in donkeys [37].

Mortality of adults in feral donkey populations is low [38]. Despite some mountain lion (*Felis concolor*) predation in Death Valley [39], there is little evidence of top-down regulation of population growth for feral donkey populations in the western United States. However, there is scant information on whether population growth is constrained by disease. Outbreaks of AHV and SEZ are known to occur based on mortalities of individuals when brought into holding facilities [40] and detection in feral donkeys from Death Valley, California [30]. However, as most feral donkey populations are not monitored at an individual level, there is little knowledge of causes of mortality and prevalence of disease.

Feral donkeys are managed on National Park Service lands in order to protect food and water sources for native species [41] and to protect cultural resources. The current lack of information on donkey presence and habitat use may impede this management. Data on donkey movements and habitat use can contribute to their management by enabling better predictions of when and where they will be found to facilitate capture for removal. Feral donkeys in the United States are managed by capturing individuals, followed by live removals to holding facilities or sanctuaries and subsequent dispersal across the country for private placement. Understanding disease prevalence in feral populations is therefore important both to determine the natural limits to population growth and to establish biosecurity measures needed after capture.

This study’s main objectives were to monitor movement and habitat selection through spatial analysis using radio telemetry and explore how they related to pathogen levels across free-roaming donkeys in the Mojave Desert in Death Valley, California. We hypothesized that donkeys would select the greenest vegetation available and proximity to water year-round, selecting north-facing slopes during hot seasons. We hypothesized that home ranges would be large to ensure access to sufficient resources. Given their prevalence and findings of prior studies, we predicted that SEZ and herpesviruses would be present in the population and hypothesized that individuals that were frequently in proximity would share infection status. As these donkeys are a non-native species present in a National Park and on military lands, we also aimed to provide information on their presence to facilitate live-capture and relocation efforts.

## 2. Materials and Methods

### 2.1. Study Area

**Figure 1:**
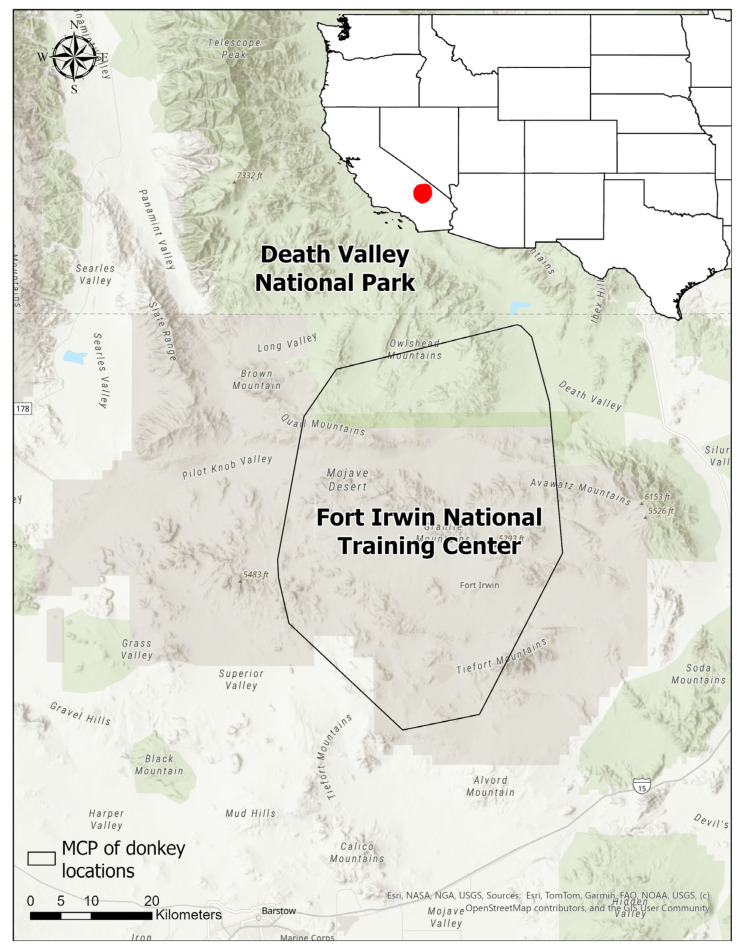
Location of study area with minimum convex polygon (MCP) of donkey radio-telemetry locations (solid black line), Death Valley National Park and Fort Irwin National Training Center, California, USA.

This study was conducted in the Mojave Desert, California, USA, in the contiguous area of Fort Irwin National Training Center and Death Valley National Park, with study animals using an area of 2600 km^2^ across both jurisdictions (Figure 1). Donkeys were managed by the jurisdictional authorities at each site, and a nongovernmental organization (Peaceful Valley Donkey Rescue (PVDR), San Angelo, TX, USA) was contracted to manage the donkey populations by capture and removal.

Temperatures in Death Valley National Park ranged between an average high of 46 °C in August and an average low of 3 °C in December, with annual average precipitation of 4.9 cm [42]. Water was available in perennial springs and ephemerally in playas and springs after precipitation. The terrain was composed of rocky mountainous ridges interspersed with salt pan desert. The habitat was desert scrub (California buckwheat [*Eriogonum fasciulatum*], white bursage [*Ambrosia dumosa*], creosote bush [*Larrea tridentata*], desert holly [*Atriplex hymenelytra*], shadscale [*A. confertifolia*], mesquite [*Prosopis glandulosa*], and blackbrush acacia [*Vachellia rigidula*]) with Joshua trees [*Yucca brevifolia*], and pinyon pine (*Pinus edulis*) and juniper (*Juniperus* spp.) at higher elevations.

### 2.2. Capture and Handling

Feral donkeys were gathered by a nongovernmental organization (NGO) contractor, and procedures were approved by public land managers across the study area. The contractor used bait trapping with alfalfa (*Medicago sativa*) to lure donkeys into a panel corral. Donkeys were subsequently separated into a chute system ending in a manual squeeze, where they were fitted with a halter and restrained for disease sampling and radio collar application. Collar application followed methods described in Schoenecker et al. [43]. Collars were placed on donkeys estimated to be adults (≥2 years old as determined by the NGO’s staff) with a body condition score of 2 to 4 (based on the scale 1 of 5 used by the Donkey Sanctuary and shown in Jerele et al. [30]). All collars were equipped with a timed remote release mechanism set to drop off after 130 weeks of deployment. Twenty-one females (jennies) and 11 males (jacks) were sampled and collared with Lotek (Lotek Wireless, Newmarket, ON, Canada) iridium GPS collars with 1 h fix rates across four collaring sessions between February 2020 and April 2021. After being collared, donkeys were released near where they were captured.

### 2.3. Disease Assays

All donkeys were assessed for nasal discharge (presence/absence), cough (presence/absence), ocular discharge (presence/absence), skin lesions (absent, swelling, hair loss, minor wound, deep wound), lameness (not lame, lame, not moving), and behavioral presentation (aggressive, alert, fearful, withdrawn, apathetic, lethargic). While restrained for collar fitting, nasal swabs were collected in virus transport media (VTM) from the left nostril, as this was more accessible in the position in which the animal was restrained. Immediately after collection, samples were transferred to the University of California Davis Veterinary Medicine PCR Laboratory in a cooler and then stored at −80 °C until testing. The median time from collection to processing was 2 days (interquartile range 2–3 days). Nasal swabs were analyzed via real-time PCR (quantitative PCR, qPCR; Table 1) at the UC Davis Veterinary Medicine PCR Laboratory for the following pathogens: *Streptococcus equi equi*, *Streptococcus equi zooepidemicus*, equine influenza type A (H3N8), equine Rhinitis A, equine Rhinitis B, Asinine Herpesvirus-2, Asinine Herpesvirus-3, Asinine Herpesvirus-5, Equine Herpesvirus-1, Equine Herpesvirus-1 (EHV-1, neuropathogenic), Equine Herpesvirus-1 (EHV-1 non-neuropathogenic), and Equine Herpesvirus-4 (EHV-4). Additional information about sample preparation, submission, and process can be found in the q-PCR diagnostic submission packet (University of California Davis Veterinary Medicine PCR Laboratory).

### 2.4. Statistical Analyses

All analyses were performed using the R statistical software (Version 4.3.2) [44]; data are available as a U.S. Geological Survey (USGS) data release [45].

#### 2.4.1. Home Range, Encounters, and Movement Rates

To minimize error and increase accuracy of results, we retained GPS locations with dilution of precision values < 10 and removed all locations with movement speeds of >4 m/s prior to analyses. Seasons were defined as hot/dry (April–October) and cool/wet (November–March) based on Karish et al. [20]. We used an automated kernel density estimator (AKDE) using the ctmm package (Version 1.2.0) [46] to estimate total and seasonal home range sizes for each donkey. We also used the ctmm package to estimate the proportion of home range overlap and number of encounters for each donkey pair. We calculated the number of encounters per pair for a sequence of buffer distances (50–500 m at 50 m intervals). We then divided the number of encounters per pair per distance buffer by the number of days they were both collared to obtain a daily encounter rate.

We used generalized linear mixed models to assess the effect of environmental variables and disease on home range size. We fitted models with a lognormal distribution and a random intercept of individual ID using the glmmTMB package (Verison 1.1.9) [47]. Environmental variables included the standard deviation of precipitation and vegetation production. We used monthly PRISM 4 km precipitation values (PRISM Climate Group 2023) to estimate the total amount of precipitation per season and then calculated the standard deviation of total seasonal precipitation across each donkey’s home range. We performed the same process for vegetation production using the enhanced vegetation index from MODIS MOD13Q1 (250 m spatial resolution, 16-day temporal resolution). The model set consisted of univariate models for each variable, plus all pairwise interactions between each variable. We ranked models using Akaike’s Information Criterion corrected for small sample sizes (AIC_c_; [48]) and assessed post hoc contrasts and generated empirical predictions using the ggeffects package (Version 1.6.0) [49]. For models within Δ2 AIC_c_, we calculated model-averaged predictions and standard errors of home range sizes. We only used female donkeys collared for >60% of the season duration (n = 17) for this analysis due to insufficient data from males.

We used a generalized linear model with a lognormal distribution to assess any differences in encounter rates between donkey pairs where each individual tested positive for AHV-5 or SEZ (the only infections found) compared to pairs where one or neither of the individuals tested positive. We used a generalized linear model with a beta distribution to test for differences in proportion of home range overlap between pairs. We used generalized linear mixed models with a lognormal distribution and a random intercept of ID to test whether AHV-5, SEZ, and season influenced movement rates of donkeys. We used the distance moved per hour as a proxy for movement rates. We removed all steps > 1 h in time and retained individuals with >500 steps. For all models, we attributed statistical significance to *p*-values ≤ 0.05.

#### 2.4.2. Environmental Covariates

We examined seasonal donkey habitat selection in response to a set of environmental covariates. We used the USGS National Elevation Dataset 1/3 arc second layers to obtain an elevation layer. We used this layer to calculate 30 m raster layers of slope and heat load index (HLI) raster layers using the Geomorphometry and Gradient Metrics Toolbox [50] within ArcGIS Pro 3.3.2 (Esri, Redlands, CA, USA). The proportion of herbaceous cover at a 30 m resolution was obtained from the National Land Cover Database shrubland fractional components layer. We applied a moving window using a 100 m buffer to the elevation, slope, HLI, and herbaceous cover rasters to account for GPS accuracy error. We used the National Hydrology Dataset to locate water bodies within the study area and calculated the Euclidean distance to water with a 30 m resolution. We used the MODIS MOD13Q1 (250 m spatial resolution, 16-day temporal resolution) to calculate the enhanced vegetation index (EVI) and instantaneous rate of green-up (IRG) following the processes of [51,52]. We scaled EVI and IRG values between 0 and 1 to represent the lowest and highest values of each variable per cell per year [52].

#### 2.4.3. Step Selection Methods

We used an integrated step selection analysis (iSSA; [53]) to assess 2nd-order habitat selection [54] during the wet and dry seasons. We extracted environmental covariate values at the end location of each step. We retained individual datasets containing >600 locations per season. We compared each used step to a set of 10 random available steps generated by modeling step lengths with a gamma distribution and turning angles with a von Mises distribution [55]. We used the amt package (Version 0.2.2.0) [56] to fit iSSA models to each individual by year combination for each season. All models contained the full set of 8 environmental covariates as well as step length, the log of step length, and the cosine of turn angles. We centered and scaled step lengths and environmental variables. We then used inverse-variance weighted regression [55] to summarize population-level response from the individual models. The inverse-variance weighting factor took the form of 1/(SE*_i_*)2, where *i* represents the corresponding environmental variable. The resulting parameter values represent the relative selection strength on the log scale (log-RSS) [57] for a 1-unit increase in the variable. Positive values indicate selection and negative values indicate avoidance. To assess how selection varied among disease status, we used the glmmTMB package (Verison 1.1.9) to fit linear mixed models weighted by inverse-variance. We ran a set of models with each environmental covariate serving as the response variable and included AHV-5 status, strep status, and year as explanatory variables. We included a random intercept of individual donkey ID. We used the ggeffects package (Version 1.6.0) to assess post hoc contrasts and generate empirical predictions for each model.

## 3. Results

### 3.1. Home Range

**Figure 2:**
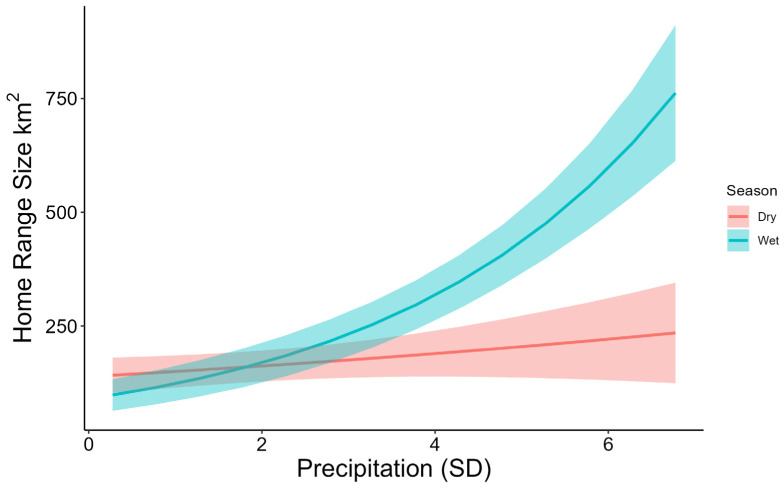
Home range size (km^2^) of feral donkeys as a function of season and standard deviation (SD) of precipitation, Death Valley, California, 2020–2022.

We fitted GPS collars to both males (n = 12) and females (n = 21). The mean dilution of precision for the GPS collar data was 2.93 (sd = 3.67). Only three of 12 collars fitted on males collected data for longer than 60 days; therefore, we report home range size solely for females. Collars were not retrieved, so the cause of failure is not fully known but is assumed to be damage-related, likely from intra-specific aggression. Mean annual home range of adult females was 301.06 km^2^ (sd = 342.85). Home range sizes were larger on average in wet seasons (Table 2), but this difference was not significant (*p* = 0.266). The best supported model explaining differences in home range sizes was the interaction between season and standard deviation of precipitation (Table 3). Home range size increased with greater standard deviation of precipitation, and this increase was greater during the wet compared to the dry season (*p* = 0.004, Figure 2). No other variable or interaction had a significant effect on home range size.

### 3.2. Disease

No animals presented with skin lesions, cough, or apathetic or lethargic behavior. One individual had minor serous nasal discharge, and three had minor serous ocular discharge. Twenty-one individuals had bacterial presence of SEZ, and 11 had viral presence of AHV-5, with eight individuals testing positive for both; all other disease assays were negative. There were no known mortalities during our study.

**Figure 3:**
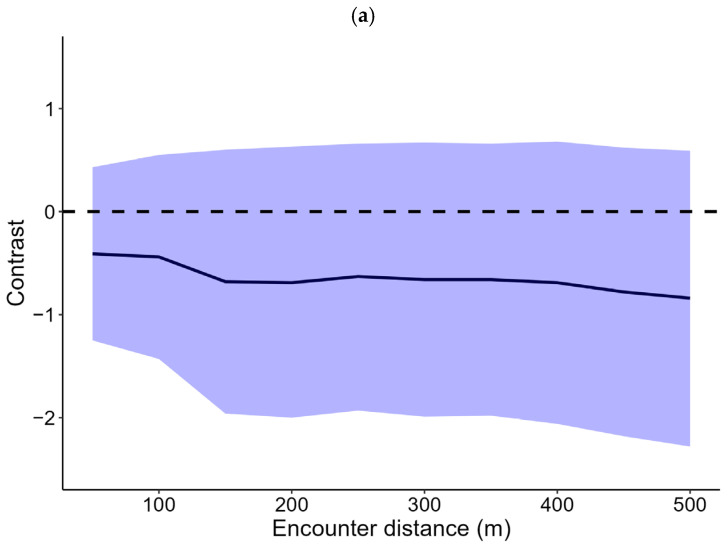

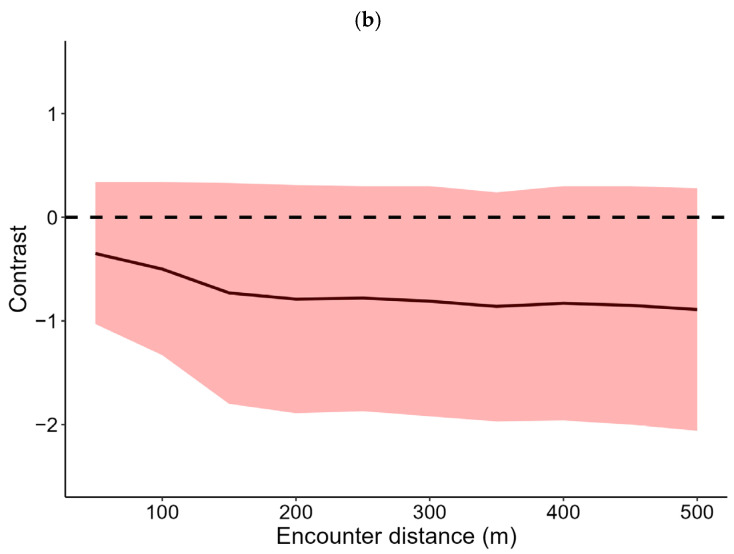
Sensitivity analyses for encounter rates between feral donkeys in the Mojave Desert, California, USA, as a function of (**a**) *Streptococcus equi zooepidemicus* (SEZ) or (**b**) asinine herpes virus 5 (AHV) infection.

Encounter rates were greater between pairs where both had evidence of AHV-5 or SEZ, but post hoc contrasts between pair types overlapped 0 at all distance buffers, indicating no difference in encounter rates among healthy or infected individuals (Figure 3). Additionally, we found no difference in the proportion of home range overlap relative to testing positive for AHV-5 (*p* = 0.935) or SEZ (*p* = 0.842). Testing positive for SEZ was the best supported model explaining differences in movement rates (Table 4) with donkeys moving farther per hour if they tested positive for SEZ; however, this difference was non-significant at the α = 0.05 level (*p* = 0.115).

### 3.3. Resource Selection

**Figure 4:**
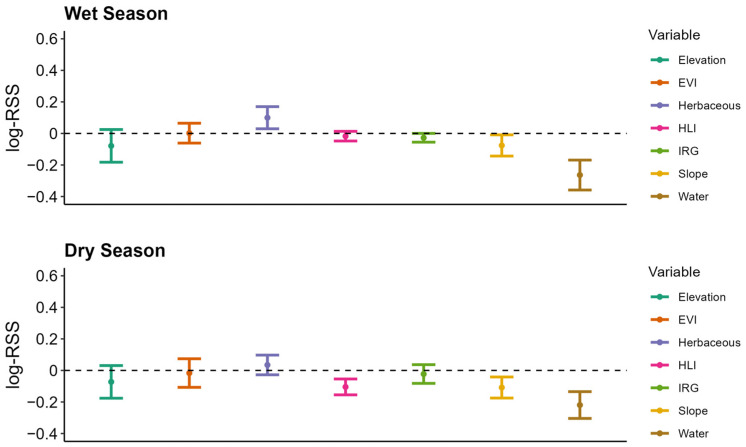
Selection indices of feral donkeys per season with 95% confidence intervals, Mojave Desert, California, USA, 2020–2022. EVI = enhanced vegetation index; HLI = heat load index; IRG = instantaneous rate of green-up.

**Figure 5:**
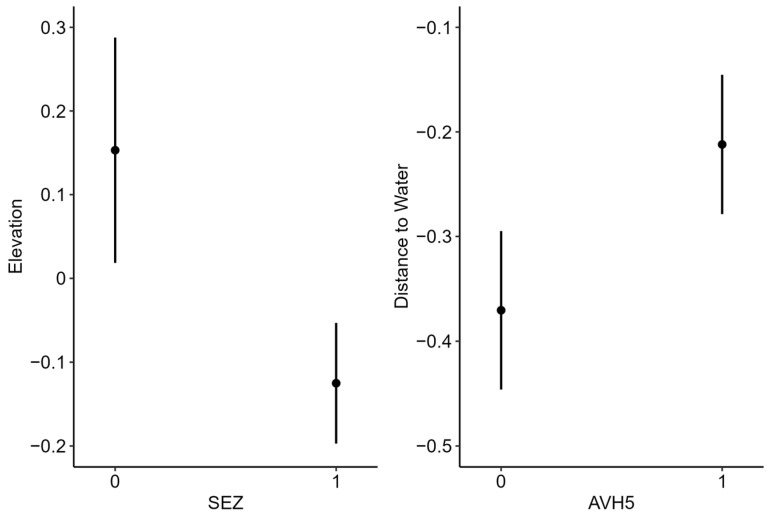
Differences in selection indices during the wet season based on *Streptococcus equi zooepidemicus* (SEZ) or asinine herpes virus 5 (AHV) infection, Mojave Desert, California, 2020–2022. 0 = not positive for disease; 1 = positive for disease.

**Figure 6:**
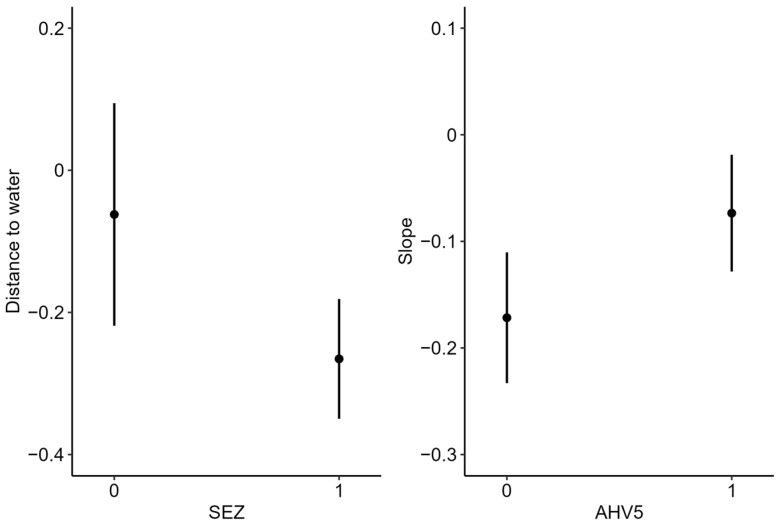
Differences in selection indices during the dry season based on *Streptococcus equi zooepidemicus* (SEZ) or asinine herpes virus 5 (AHV) infection, Mojave Desert, California, 2020–2022. 0 = not positive for disease; 1 = positive for disease.

During the wet season, female donkeys selected flatter slopes, greater herbaceous cover, and closer proximity to water (Figure 4). Proximity to water had the greatest effect on selection, followed by herbaceous cover and slope (Figure 4). During the dry season, donkeys selected closer proximity to water, flatter slopes, and lower heat loads (Figure 4). Proximity to water again had the strongest effect on selection, followed by slope and heat load index. During the wet season, donkeys testing positive for SEZ selected lower elevations, and donkeys with evidence of AHV-5 selected areas farther from water (Figure 5). Comparatively, during the dry season, donkeys with evidence of SEZ selected closer proximity to water, and donkeys with evidence of AHV-5 selected steeper slopes (Figure 6).

## 4. Discussion

The home ranges observed were comparable to those estimated by Karish et al. [20] in the same habitat of the Mojave Desert. Karish et al. [20] found home ranges of around 254 km^2^, and we found home ranges of around 159 km^2^ and 318 km^2^, depending on season. The apparent difference between seasons, although non-significant, is where our results differ from those of Karish et al. [20], who found no effect of season. This difference between our findings could be related to our larger sample size of collared individuals and the fact that we sampled animals in the undisturbed habitat of Death Valley National Park in addition to Fort Irwin National Training Center. Donkeys on Fort Irwin selected areas closer to urban areas, where resources are more consistent due to irrigation [20].

Donkeys are an arid-adapted species, being able to survive dehydration of up to 30% of their body weight and rehydrate sufficiently to return blood constituents to normal levels within 3–60 min of drinking [58,59,60]. In addition to sweating, donkeys can maintain homeothermy by respiratory evaporative heat loss [61]. Despite this, our results demonstrated the importance of water availability for this species. In this study we found that donkeys selected areas closer to water sources year-round, and in areas with differing amounts of precipitation during the wet season, donkeys expanded their space use to include new water sources and perhaps find more forage. An increase in home range size with a greater standard deviation of precipitation across a home range indicates that donkeys could travel more widely during times of precipitation when temperatures were cooler and more surface water was available, as observed in another population of feral donkeys in central Utah [21]. Both donkeys and wild ass species (African wild ass and Asiatic wild ass, *E. hemionus*) visit known water sources less frequently during wetter seasons than dry seasons [20,62,63,64,65].

Contrary to our hypotheses, donkeys did not select greener vegetation during the dry season or wet season. Our results indicate that lower heat loads are relatively more important than green vegetation production for predicting space use of donkeys during the hot/dry season in Death Valley. The hot/dry season is physiologically stressful for working donkeys in Nigeria [61], with donkeys seeking shelter from solar radiation as much as they sought refuge from high temperatures [22]. Feral donkeys have been observed to use aspect and availability of shade to shelter from summer temperatures [21], with Asiatic wild asses using high elevations and northern exposures [62]. Moreover, vegetation is sparsely distributed in the Mojave Desert, so the spatial scale of our greenness metric (250 m) may be too large to adequately reflect variation in actively photosynthesizing plant production, leading to our finding of neither selection nor avoidance of EVI during either season.

Although there was no effect on home range size, we did find differences in resource selection indices relative to whether donkeys showed presence of SEZ or AHV-5. Donkeys with evidence of SEZ selected lower elevations relative to non-infected conspecifics in the wet season and closer proximity to water in the dry season. Comparatively, donkeys with evidence of AHV-5 selected more rugged terrain in the dry season and farther distances to water during the wet season. Presence of pathogen genetic material in nasal swabs does not provide information about current active replication and infectious shedding of SEZ or activation status of AHV-5, and single sampling does not allow us to make conclusions about shedding or disease transmission. However, some strains of herpes virus, such as EHV-1 and 4, can be spread through aerosol transmission from nasal discharge, with fomites remaining infectious on wood, straw, and iron for up to 7 days [34]. Alternatively, SEZ is transmitted through close contact between individuals; although less aggregation of individuals is expected near water sources in the wet season, use of these areas could promote transmission among the population.

Infection by both AHV-5 and SEV was inapparent when donkeys were closely observed, with all animals appearing alert and in good condition. In arid environments donkeys have a fission–fusion social system [9,10]. In these social systems there are no permanent social associations between adults, but individuals come together and separate as they independently forage; however, the social network of donkeys is saturated, as individuals are well connected with many other individuals in the population [9]. Where individuals have a high number of social partners that interact with other social partners, as observed in a Sonoran Desert feral donkey population [9], there is an increased probability of disease spread among a population. In this study we did not find a greater risk of both members of a dyad being infected when they had higher encounter rates and found no effect of overlapping home ranges on risk of infection.

Limitations of our study were our relatively small sample size and our lack of data on males. A larger sample across both sexes would have allowed for greater inference of donkey movements and habitat selection and may have revealed more differences between donkeys with and without evidence of SEZ and AHV-5. We used single sampling events for PCR detection of disease, but as we had a small sample size and few clinical signs, use of serology for antibody detection to demonstrate pathogen exposure may have been more fruitful.

Both SEZ and AHV-5 detected in feral donkeys in Death Valley are highly infectious among equids, with AHV-5 having the potential to transmit across species. Donkeys can act as disease reservoirs: while they may be susceptible to diseases from horses [24], they can also transmit them. Asinine herpes virus 5 (AHV-5) has the potential to co-infect horses, presenting as equine multinodular pulmonary fibrosis [32,66]. *Streptococcus equi zooepidemicus* has the potential to affect all equids but can also affect livestock, carnivores, and humans, causing bacteremia, meningitis, and arthritis in people [26]. As donkeys may not demonstrate clinical signs of disease, managers may implement biosecurity measures, such as disinfecting equipment when handling or transporting these animals from a feral to a domestic setting. Although a large proportion of individuals may be colonized with SEZ or latent carriers of AHV-5, we had no indication that this was controlling the population during the study. It is possible that disease may have fitness costs on infected individuals, slowing population growth, and that stronger population-level effects are shown during times of stress.

## 5. Conclusions

This work contributes to knowledge of the ecology of donkeys in the southwestern United States, a species that has been relatively little studied compared to feral horses and native ungulates. Despite donkeys being arid-adapted, we showed that their movements, ranges, and habitat use were shaped by precipitation and the presence of water. Our results imply that while there is the potential for disease to spread among the population based on donkey social structure, this is mediated by low prevalence of the disease and different habitat use by infected animals. Donkeys are not protected under the Free-Roaming Wild Horse and Burro Act while on National Park or military jurisdictions, and managers may aim to remove them as a non-native species. Our results highlight that bait trapping may be most successful near water sources year-round and in areas of herbaceous vegetation in the wet season and areas with more shade in the dry season.

## Figures and Tables

**Table 1:** Target genes used for qPCR-assay pathogen load testing at the University of California Davis Veterinary Medicine PCR Laboratory.

Assay Name	Gene, NCBI ^1^ Accession Number	Assay Location (bp)	Amplicon Length (bp)
Asinine Herpesvirus 2	Polymerase, EU165547	100	81
Asinine Herpesvirus 3	Glycoprotein B, U24184	740	145
Asinine Herpesvirus 5	Polymerase, AY054993	600	64
Equine Herpesvirus 1	Glycoprotein B, NC_001491	400	89
Equine Herpesvirus 1, neuropathogenic	ORF 30, KF644574	200	92
Equine Herpesvirus 1, non-neuropathogenic	ORF 30, KX101095	200	92
Equine Herpesvirus 4	Glycoprotein B, AF030027	440	77
*Streptococcus equi* subspecies *equi*	M Protein, AF012927	150	185
*Streptococcus equi* subspecies *zooepidemicus*	ITS, EU860336	80	88
Influenza A (H3N8)	Hemagglutinin Precursor, EF541443	350	200
Equine rhinitis A virus	RNA polymerase, X96870	150	111
Equine rhinitis B virus	RNA polymerase, X96871	350	87
Glyceraldehyde-3-phosphate dehydrogenase	GAPDH, AF097179	60	105

^1^ NCBI: National Center for Biotechnology Information.

**Table 2:** Mean, median, and standard deviation (SD) of home range sizes for feral donkeys in the Mojave Desert, California, USA, during the dry (April–October) and wet (November–March) seasons, 2020–2022.

Season	Number of Individuals	Mean	Median	SD
Dry	14	159.35	63.89	212.43
Wet	12	318.37	111.40	417.65
Dry 2020	9	153.63	39.06	187.37
Dry 2021	10	94.90	72.80	74.85
Wet 2021	8	186.59	94.93	231.54
Wet 2022	5	529.22	480.88	582.37

**Table 3:** Model selection table of home range sizes of feral donkeys in the Mojave Desert, California, USA, as a function of *Streptococcus equi zooepidemicus* (SEZ) or asinine herpes virus 5 (AHV) infection, precipitation, vegetation production (EVI), and season. Number of parameters (K), Akaike’s Information Criterion corrected for small sample sizes (AIC_c_), and weight (w_i_) are given for each model.

Model	K	AIC_c_	ΔAIC_c_	w_i_
Precipitation * Season	6	409.2	0.00	0.40
Precipitation	4	409.8	0.62	0.29
Precipitation * EVI	6	410.3	1.13	0.22
Precipitation * SEZ	6	413.4	4.25	0.05
Precipitation * AHV	6	414.0	4.81	0.04
AHV	4	419.7	10.50	0.00
Season	4	420.3	11.09	0.00
SEZ * Season	6	421.4	12.21	0.00
EVI	4	421.4	12.25	0.00
SEZ	4	421.7	12.49	0.00
EVI * Season	6	422.4	13.21	0.00

**Table 4:** Model selection table of hourly step lengths of feral donkeys in the Mojave Desert, California, USA, as a function of *Streptococcus equi zooepidemicus* (SEZ) or asinine herpes virus 5 (AHV) infection and season. Number of parameters (K), Akaike’s Information Criterion corrected for small sample sizes (AIC_c_), and weight (w_i_) are given for each model.

Model	K	AIC_c_	ΔAIC_c_	w_i_
SEZ	4	4,037,273	0.00	0.52
AHV	4	4,037,275	1.87	0.21
Season	4	4,037,275	2.24	0.17
SEZ * Season	6	4,037,277	4.00	0.07
AHV * Season	6	4,037,279	5.87	0.03

## Data Availability

Data are available as a USGS data release [45] https://doi.org/10.5066/P178ABUK.
